# Closed-Loop Torque and Kinematic Control of a Hybrid Lower-Limb Exoskeleton for Treadmill Walking

**DOI:** 10.3389/frobt.2021.702860

**Published:** 2022-01-20

**Authors:** Chen-Hao Chang, Jonathan Casas, Steven W. Brose, Victor H. Duenas

**Affiliations:** ^1^ Department of Mechanical and Aerospace Engineering, Syracuse University, Syracuse, NY, United States; ^2^ Department of Physical Medicine and Rehabilitation, SUNY Upstate Medical University, Syracuse, NY, United States; ^3^ Spinal Cord Injury and Disabilities Service, Syracuse VA Medical Center, Syracuse, NY, United States

**Keywords:** nonlinear systems, torque and kinematic control, Lyapunov methods, functional electrical stimulation (FES), Lower-limb exoskeleton

## Abstract

Restoring and improving the ability to walk is a top priority for individuals with movement impairments due to neurological injuries. Powered exoskeletons coupled with functional electrical stimulation (FES), called hybrid exoskeletons, exploit the benefits of activating muscles and robotic assistance for locomotion. In this paper, a cable-driven lower-limb exoskeleton is integrated with FES for treadmill walking at a constant speed. A nonlinear robust controller is used to activate the quadriceps and hamstrings muscle groups *via* FES to achieve kinematic tracking about the knee joint. Moreover, electric motors adjust the knee joint stiffness throughout the gait cycle using an integral torque feedback controller. For the hip joint, a robust sliding-mode controller is developed to achieve kinematic tracking using electric motors. The human-exoskeleton dynamic model is derived using Lagrangian dynamics and incorporates phase-dependent switching to capture the effects of transitioning from the stance to the swing phase, and vice versa. Moreover, low-level control input switching is used to activate individual muscles and motors to achieve flexion and extension about the hip and knee joints. A Lyapunov-based stability analysis is developed to ensure exponential tracking of the kinematic and torque closed-loop error systems, while guaranteeing that the control input signals remain bounded. The developed controllers were tested in real-time walking experiments on a treadmill in three able-bodied individuals at two gait speeds. The experimental results demonstrate the feasibility of coupling a cable-driven exoskeleton with FES for treadmill walking using a switching-based control strategy and exploiting both kinematic and force feedback.

## 1 Introduction

The loss of motor and sensory function associated with spinal cord injury (SCI) results in limited mobility, lack of independence, and diminished quality of life (Kirshblum and Lin, 2018; Hornby et al., 2020). Restoring and improving the ability to walk is a top priority for individuals with paralysis, whose locomotion is affected by muscle weakness, impaired postural stability and reduced leg coordination Anderson (2004). Robotic exoskeletons assist individuals with paralysis to improve their gait kinematics, cardiorespiratory and metabolic responses, balance, and mobility (Field-Fote and Roach, 2011; Yang et al., 2015; Ramanujam et al., 2018; Kressler et al., 2018; Sale et al., 2018; Kressler and Domingo, 2019; Hornby et al., 2020; Hong et al., 2020). However, exoskeletal-assisted walking in isolation faces challenges in improving muscle capacity and reinforcing the activation of paralyzed muscles during locomotion (Edgerton et al., 2001; Field-Fote and Roach, 2011). Alternatively, a neuromuscular control approach such as functional electrical stimulation (FES) evokes muscle contractions to replace or assist human volition (Reed, 1997; Peckham and Knutson, 2005). FES applies electrical stimuli across skeletal muscles and can yield benefits such as improved muscle strength, blood flow, bone mineral density, and range of motion (Reed, 1997; Peckham and Knutson, 2005; Doucet et al., 2012). However, isolated control of FES for walking without robotic assistance is challenging due to the nonlinear muscle activation rate and accelerated onset of muscle fatigue (Lynch and Popovic, 2008; Bickel et al., 2011; Downey et al., 2017). A hybrid approach integrating robotic exoskeletons and FES (termed hybrid exoskeletons) Ho et al. (2014); Chang et al. (2015), Chang et al. (2017 SR.) provides the benefits of actively stimulating paralyzed muscles and exploits the robot’s torque reliability to yield repetitive motion. Furthermore, hybrid exoskeletons can contribute to delay the onset of muscle fatigue by reducing the muscle stimulation duty cycle and extend walking endurance. Innovations for the control design and analysis are needed to achieve an effective integration of FES with robotic exoskeletons that interface the human body with different actuation mechanisms.

Hybrid exoskeletons provide postural support, coordinate motion across multiple joints, and apply bursts of electrical stimulation. Several hybrid exoskeletons have incorporated direct joint actuation and implemented closed-loop controllers for the powered machines and FES (Ha et al., 2016; Alibeji et al., 2018a). Hybrid orthoses have been designed to lock and unlock leg joints as a function of the gait cycle to provide upright stability and leg assistance using postural controllers (Kobetic et al., 2009). A hybrid neuroprosthesis (HNP) evaluated a finite state machine controller to coordinate stimulation and exoskeleton inputs for stepping Chang SR. et al. (2017). A HNP with variable-constraint hip mechanisms and neuromuscular stimulation reduced forward lean during walking and improved gait speed To et al. (2014). A hybrid system integrating an exoskeleton to actuate hip and knee joints, and implanted neural stimulation has been developed to increase gait speed in individuals with SCI Nandor et al. (2021). Cooperative control between motor and muscle loops has been developed to minimize the motor torque contribution and maximize the muscle-generated joint torques *via* surface stimulation (Ha et al., 2016). A position-based controller combining neural networks and classical adaptive control was designed to synchronize a robotic manipulator and FES during assisted leg extension (Alibeji et al., 2017). A closed-loop adaptive control design using iterative learning and neural networks was developed to distribute the control between FES and electric motors to perform sit-to-stand tasks (Molazadeh et al., 2021). Switched control between two modes was developed for a wearable exoskeleton with FES to address nonlinearities and uncertainties in the overall system (Sheng et al., 2021). A controller inspired by the principle of synergies was used to address the problem of actuator redundancy in simulation to control muscles *via* FES and electric motors (Alibeji et al., 2015). In Alibeji et al. (2018a,b), a muscle synergy-based controller was developed to control muscles and motors accounting for the muscle activation dynamics and the inherent electromechanical delay of muscles. The results in Alibeji et al. (2018a,b) included a rigorous Lyapunov-based stability analysis and experiments with one able-bodied individual and one participant with incomplete SCI. Despite the advances in hybrid exoskeletons, technical innovations are needed to improve the obtained walking speeds and distances (Chang et al., 2020) and yield more natural and compliant interactions for people with varying levels of volition.

Differently from exoskeletons with direct joint motor actuation, soft exoskeletons use wearable garments and Bowden cables to enable human transparent movements for walking and running (Witte et al., 2015, Witte et al., 2017; Collins et al., 2015; Asbeck et al., 2014; Park et al., 2020; Di Lallo et al., 2021). Cable-driven exoskeletons traditionally offload electric motors, gear transmissions, and other components away from the human body. These design features make cable-driven exoskeletons desirable to be interfaced with individuals with varying levels of volition during walking training. Soft wearable exoskeletons and ankle emulators have reduced the metabolic energy consumption during walking by optimizing control parameters in able-bodied individuals and stroke survivors (Zhang et al., 2017; Ding et al., 2018; Witte et al., 2020). Soft exoskeletons have implemented a human-in-the-loop paradigm (Ding et al., 2018; Siviy et al., 2020) demonstrating improvements in walking speed and distance for post-stroke individuals. Cable-driven exoskeletons also hold the potential to be used for function restoration during gait rehabilitation including their combination with FES. Lighter exoskeletons can provide less resistance to muscle effort and could potentially reduce the metabolic costs of walking compared to more rigid exoskeletons Chang S. R. et al. (2017). Integrating the system-level benefits of cable-driven exoskeletons and muscle-driven benefits of FES can provide customized walking behaviors. However, challenges remain to design and evaluate feasible and intuitive control strategies for cable-driven exoskeletons and FES during walking, while developing rigorous control analysis for the multi-joint hybrid system.

A fundamental research question for the development of hybrid exoskeletons is how to allocate or segregate the control design for the powered actuators and muscles (Alibeji et al., 2018a; Alibeji et al., 2018b; Ha et al., 2016). Kinematic tracking has been the primary control objective for rehabilitation devices and machines that combine FES and powered actuation, where the desired trajectories can be tracked by muscles, electric motors (i.e., the machine or robot) or both during walking and cycling (Alibeji et al., 2018b; Duenas et al., 2019; Cousin et al., 2021). Recently, torque tracking objectives have been developed for motorized FES-cycling using admittance-based or impedance-based strategies with a Lyapunov-based analysis (Chang and Duenas, 2019; Duenas et al., 2020; Cousin et al., 2020). Cable-driven exoskeletons allow for the design of force feedback controllers, by including force transducers, as a strategy to adjust the cable tension and influence joint kinematics and kinetics. In particular, muscular and joint stiffness are essential for leg coordination and regulation of posture (Duysens et al., 2000; Nichols, 2002). Stiffness control is motivated for human-machine interaction for its ability to absorb shock, robustness to perturbations, efficiency to release and store energy, and safety (Vanderborght et al., 2013; Grioli et al., 2015; Keemink et al., 2018). Stiffness control has been widely used in industrial manufacturing machines, grasping for robotic hands Garate et al. (2018), upper-limb exoskeletons (Li et al., 2018), ankle actuators (Moltedo et al., 2019), and surgical manipulators (Mahvash and Dupont, 2011). Stiffness controllers traditionally use impedance-based models to generate interaction torques based on changes in the joint kinematics. Gait training was performed using an admittance controller in a robot that converts desired assistance to joint trajectories and stiffness profiles (Meuleman et al., 2016). A hybrid FES-exoskeleton cooperative strategy exploited a torque field with stiffness and damping for the motor control, while kinematic controllers were designed for the muscles (Del-Ama et al., 2014). The motivation in this paper is to exploit the ability of the cable-driven exoskeleton to adjust the joint stiffness and design FES controllers for muscles during walking.

In this paper, kinematic and torque tracking controllers are designed to activate the electric motors of a lower-limb cable-driven exoskeleton and muscles *via* FES to achieve treadmill walking at a constant speed. The hybrid exoskeleton and human are modeled as a four-link bipedal walking system with state-dependent switched dynamics to describe the gait phase transitions from stance phase to swing phase, and vice versa. The leg dynamics are modeled as a switched system to characterize the pendulum dynamics within the swing phase and the inverted pendulum dynamics within the stance phase, and their ongoing transitions during walking. The quadriceps and hamstrings muscle groups are electrically stimulated using a nonlinear robust kinematic feedback controller to guide the knee joints through their desired joint angles, while the electric motors adjust the cable tension to achieve a desired torque using a stable stiffness model. The electric motors provide torque assistance about the hip joints to track the desired hip joint angles. The bipedal walking dynamics include low-level switching to determine the active muscles and electric motors to achieve flexion and extension motion for the knee and hip joints. A Lyapunov-based approach is developed to ensure exponential tracking of the kinematic and torque closed-loop systems. Experimental results in three able-bodied individuals are presented describing the feasibility of the control methods. A discussion on the obtained experimental results and the future work are described.

## 2 Dynamic Model

### 2.1 Human-Exoskeleton Dynamics

**FIGURE 1:**
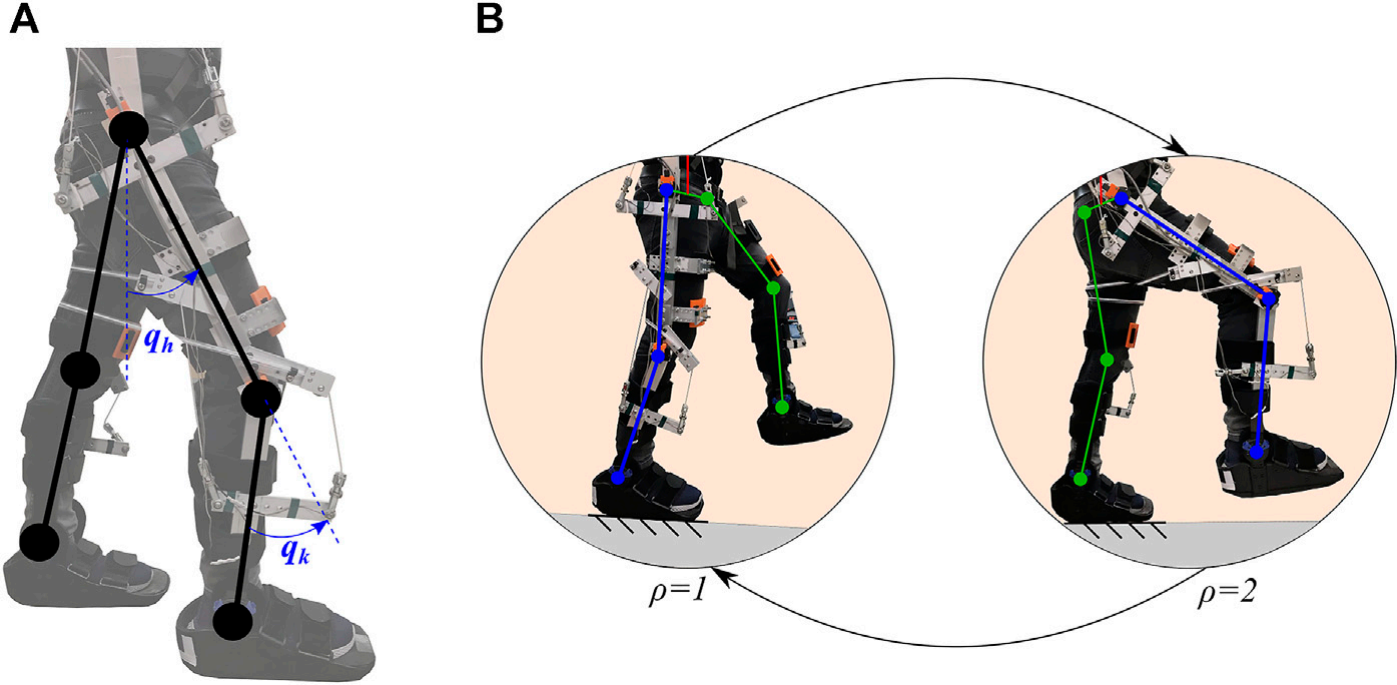
Schematic of the human-exoskeleton system with switching dynamics. **(A)** The knee joint angle *q*_*k*_ and hip joint angle *q*_*h*_ of the right leg are depicted in an initial standing position. **(B)** The switching dynamics are illustrated using the stance leg as reference (i.e., support leg). The subsystem *ρ* = 1 denotes when the right leg is in the stance phase and the left leg is in the swing phase. The subsystem *ρ* = 2 denotes when the left leg is in the stance phase and the right leg is the swing phase.

The hybrid exoskeleton and a human can be modeled as a four-link bipedal walking system in the sagittal plane with a switching Euler-Lagrange model as

$$M_{\rho }\left(q\right)\ddot{q}+C_{\rho }\left(q,\dot{q}\right)\dot{q}+G_{\rho }\left(q\right)+P_{\rho }\left(q,\dot{q}\right)+d_{\rho }\left(t\right)=\tau_{E}\left(q,\dot{q},t\right)+\tau_{M}\left(q,\dot{q},t\right),$$ (1)

where the subscript ρ∈R≜{1,2} denotes the index of the switching dynamics using the stance leg as the reference, such that *ρ* = 1 denotes the right leg as the stance leg and *ρ* = 2 denotes the left leg as the stance leg, as illustrated in Figure 1. The joint angle vector is defined as $q≜{\left[q_{rk},q_{lk},q_{rh},q_{lh}\right]}^{T}:R_{\geq t_{0}}\to R^{4}$ denoting the measurable joint angles (i.e., right (r), left (l), knee joint (k), and hip joint (h), respectively), $\dot{q}:R_{\geq t_{0}}\to R^{4}$ and $\ddot{q}:R_{\geq t_{0}}\to R^{4}$ denote the measurable joint angular velocities and unmeasurable joint angular accelerations, respectively, and $t_{0}\in R_{>0}$ is the initial time; $M_{\rho }:R^{4}\to R_{>0}^{4\times 4}$ denotes the combined human-exoskeleton inertia; $C_{\rho }:R^{4}\times R^{4}\to R^{4\times 4}$ and $G_{\rho }:R^{4}\to R^{4}$ denote the Centripetal-Coriolis and gravitational effects, respectively; $P_{\rho }:R^{4}\times R^{4}\to R^{4}$ denotes the damping and viscoelastic effects; and $d_{\rho }:R_{\geq t_{0}}\to R^{4}$ denotes lumped disturbances applied to the system by the legs (e.g., involuntary forces and muscle spastic contractions), ground reaction effects, and any other unmodeled effects present in the system.

**FIGURE 2:**
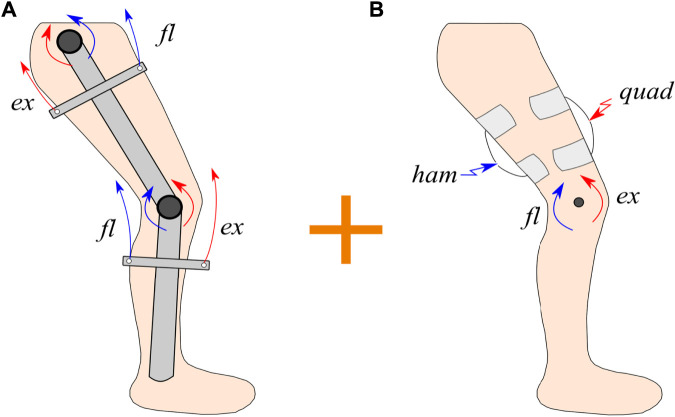
Schematic of the hybrid exoskeleton actuation for walking. **(A)** The actuation of the cable-driven exoskeleton is illustrated, where a couple of flexor and extensor cables on each joint are tensioned to provide torque about the joints. **(B)** The FES applied to the quadriceps (quad) and hamstrings (ham) muscle groups to generate torque about the knee joint is depicted.

The torque inputs in Eq. 1 include $\tau_{E}:R^{4}\times R^{4}\times R_{\geq t_{0}}\to R^{4}$ , which denotes the torque applied by electric motors, and $\tau_{M}:R^{4}\times R^{4}\times R_{\geq t_{0}}\to R^{4}$ , which denotes the active torque produced by active muscle contractions *via* FES. Hence, the hybrid exoskeleton integrates electric motors and FES applied on the muscles to actuate the hip and knee joints as illustrated in Figure 2. The cable-driven mechanism provides tension to flexor (*fl*) and extensor (*ex*) cables using electric motors. Similarly, FES is applied to the hamstrings (ham) and quadriceps (quad) muscle groups to achieve knee flexion and extension, respectively. Electric motors provide torque about both knee and hip joints (Figure 2A). FES evokes muscle contractions to generate torque about the knee joint since the quadriceps and hamstrings are assumed to produce torque only about the knee joint (i.e., negligible hip coactivation). (Figure 2B). The activation of hip flexors and extensors is challenging using surface FES Alibeji et al. (2018a), hence these muscles are not stimulated and do not contribute to generate torque about the hip joint. The muscle and motor torque inputs are described in the next subsection.

### 2.2 Actuator Input Switching

**FIGURE 3:**
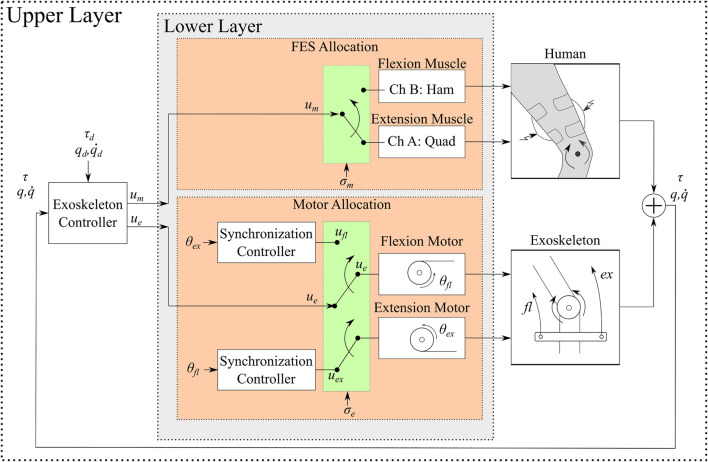
Block diagram of the two-layer control scheme. The upper layer is the joint feedback loop to generate the muscle and motor control inputs *u*_*m*_ and *u*_*e*_, respectively. The muscle and motor inputs can exploit kinematic or torque feedback. The lower layer allocates the upper layer control inputs *u*_*m*_, *u*_*e*_ to individual muscles and motors. The allocation is dictated by the switching signals *σ*_*m*_, *σ*_*e*_ to apply FES and electric currents, respectively. Within the lower motor layer, a synchronization controller is designed to maintain suitable cable tension and avoid cable slackness.

The lower-limb hybrid exoskeleton actuates joints using electric motors that drive customized cable-driven mechanisms combined with FES applied to the muscles. The allocation of the control commands to a subset of actuators (i.e., motors and muscles) is needed to yield adequate leg coordination. Hence, a two-layer scheme is designed and illustrated in Figure 3. The upper layer is the exoskeleton joint control loop, where the desired tracking objectives (i.e., kinematic and stiffness tracking) are achieved by the designed muscle and motor control inputs *u*_*m*_, *u*_*e*_, respectively, subsequently defined. The lower layer allocates the muscle and motor inputs *u*_*m*_, *u*_*e*_, computed in the upper layer, to individual muscles and motors. Such allocation is achieved by means of the switching signals *σ*_*m*_, *σ*_*e*_ that activate muscles and motors, respectively, to achieve flexion or extension. Within the lower layer for the motors, a synchronization controller is designed to prevent a slacking behavior in the cables and improve the response time of the motors. Figure 3 depicts the block diagram of the upper and lower control layers. The design and stability analysis of the synchronization motor controller is described in Chang et al. (2021). This controller is implemented for each pair of motors that actuate any given joint. During the implementation of the synchronization motor controller one motor (lead motor) receives the upper layer input *u*_*e*_, whereas the other motor (follower motor) receives the synchronization control input. This synchronization control input is designed to track a desired motor angular position to maintain appropriate tension and reduce cable slackness (Chang et al., 2021). The torque produced by motors and muscles can be defined as

$$\begin{array}{ll} \tau_{E}\left(q,\dot{q},t\right) & ≜\overset{8}{\underset{e=1}{\sum }}B_{e}\left(q,\dot{q}\right)\sigma_{e}\left(t\right)u_{e}\left(t\right), \end{array}$$ (2)

$$\begin{array}{ll} \tau_{M}\left(q,\dot{q},t\right) & ≜\overset{4}{\underset{m=1}{\sum }}B_{m}\left(q,\dot{q},t\right)\sigma_{m}\left(t\right)u_{m}\left(t\right), \end{array}$$ (3)

where the subscript e∈E={1,2,…,8} denotes the motor index, and m∈M={1,2,3,4} denotes the muscle group index as illustrated in Table 1. The unknown individual motor control effectiveness is denoted as $B_{e}:R^{4}\times R^{4}\to R_{>0}^{4\times 4}$ , and the individual motor current inputs are denoted as $u_{e}:R_{\geq t_{0}}\to R^{4},\;\forall e\in E$ . The unknown individual muscle control effectiveness is denoted by $B_{m}:R^{4}\times R^{4}\times R_{\geq t_{0}}\to R_{>0}^{4\times 4}$ and the individual muscle stimulation inputs are denoted by $u_{m}:R_{\geq t_{0}}\to R^{4},\;\forall m\in M$ . The switching signals are defined as the piecewise constant functions *σ*_*e*_ ∈ {0, 1} and $\sigma_{m}\in \left\{0,1\right\},\forall e\in E,m\in M$ .

**TABLE 1:** Flexion and extension motor indices for right and left hip and knee joints (top). Quadriceps and hamstrings muscles indices for right and left leg (bottom).

	Right knee	Left knee	Right hip	Left hip
Extension Motor	1	3	6	8
Flexion Motor	2	4	5	7

	Right leg	Left leg
Quadriceps	1	3
Hamstrings	2	4

The following properties are exploited in the subsequent control design and stability analysis.

**Property 1** The inertia matrix M_ρ_(q) is positive definite and symmetric, and satisfies the inequalities $c_{m}{\left\|\xi \right\|}^{2}\leq \xi^{T}M_{\rho }\left(q\right)\xi \leq c_{M}{\left\|\xi \right\|}^{2}$ , $\forall \xi \in R^{4}$ , where c_m_ and c_M_ are known positive constants, ∀ρ∈R L.Lewis et al. (2004).

**Property 2** The inverse of the inertia matrix M_ρ_(q) is bounded as $\frac{1}{c_{M}}I\leq M_{\rho }^{-1}\left(q\right)\leq \frac{1}{c_{m}}I$ , ∀ρ∈R , where I is the identity matrix L.Lewis et al. (2004).

**Property 3** $\left\|C_{\rho }\left(q,\dot{q}\right)\right\|\leq c_{c}\left\|\dot{q}\right\|$ , ∀ρ∈R , where c_c_ is a known positive constant L.Lewis et al. (2004).

**Property 4** $\left\|G_{\rho }\left(q\right)\right\|\leq c_{g}$ , ∀ρ∈R , where c_g_ is a known positive constant L.Lewis et al. (2004).

**Property 5** $\left\|P_{\rho }\left(q,\dot{q}\right)\right\|\leq c_{p1}+c_{p2}\left\|\dot{q}\right\|$ , ∀ρ∈R , where c_p1_ and c_p2_ are known positive constants (Ferrarin and Pedotti, 2000; Sharma et al., 2009; Schauer et al., 2005).

**Property 6** The lumped kinematic switching control effectiveness is a diagonal matrix and is bounded as ${\underline{B}}_{\kappa }{\left\|\xi \right\|}^{2}\leq \xi^{T}B_{\kappa }\xi \leq {\bar{B}}_{\kappa }{\left\|\xi \right\|}^{2}$ , $\forall \xi \in R^{4}$ , where ${\underline{B}}_{\kappa }$ and ${\bar{B}}_{\kappa }$ are known positive constants.

**Property 7** The lumped stiffness switching control effectiveness is a diagonal matrix and is bounded as ${\underline{B}}_{s}{\left\|\zeta \right\|}^{2}\leq \zeta^{T}B_{s}\zeta \leq {\bar{B}}_{s}{\left\|\zeta \right\|}^{2}$ , $\forall \zeta \in R^{2}$ , where ${\underline{B}}_{s}$ and ${\bar{B}}_{s}$ are known positive constants.

**Assumption 1** $\left\|d_{\rho }\left(t\right)\right\|\leq c_{d}$ , ∀ρ∈R , where *c*_*d*_ is a known positive constant.

## 3 Control Development

The control design is segregated for the stance and swing phases of walking. To absorb the foot impact and guarantee trunk support during early stance, the stiffness in the knee joint is increased and knee extensor activity is modulated (Neptune et al., 2008), which ultimately contributes to enable body propulsion and initiate swing. On the other hand, during the swing phase, leg stiffness is reduced to increase compliance and allow smooth knee joint kinematics and prepare for heel strike (i.e., contact with the ground). Hence, the knee joint stiffness contributes for shaping the leg dynamics along with the hip joint that assist body propulsion and preserve the rhythmic walking motion (Perry, 1992; Duysens et al., 2000). Inspired by such joint behaviors, a multiple control objective is developed in this paper to adjust the joint stiffness and kinematics on both phases, while ensuring a stable rhythmic walking motion.

**FIGURE 4:**
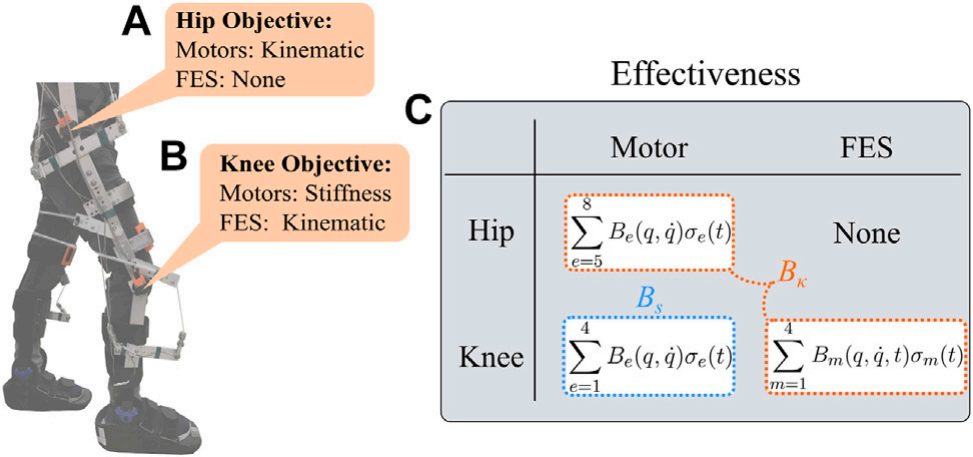
Schematic of the kinematic (*κ*) and stiffness (*s*) control tracking objectives. **(A)** The hip joint is actuated only by the electric motors to achieve kinematic tracking (i.e., no FES is applied). **(B)** The knee joint is controlled to achieve both control objectives: electric motors track the stiffness objective and the muscles track the kinematic objective. **(C)** The table shows the control effectiveness matrices associated with the kinematic and stiffness control objective, *B*_*κ*_ and *B*_*s*_, respectively.

Two control objectives are developed as depicted in Figure 4. The first objective is to design kinematic controllers (*κ*) to track knee and hip joint trajectories. A pair of electric motors achieve the kinematic tracking objective for the hip joints, whereas FES applied to the quadriceps and hamstrings achieve kinematic tracking for the knee joints. The second objective is to design a knee joint stiffness controller (*s*) throughout the gait cycle using the electric motors that actuate the knee joints. Since the electric motors and FES cooperate to achieve both control objectives, the control effectiveness matrices can be segregated for the kinematic and stiffness control objectives as depicted in Figure 4C, where the lumped effectiveness *B*_*κ*_ and *B*_*s*_ are defined for the kinematic and stiffness control loops, respectively. The control design for each objective is developed in the subsequent subsections. A robust control technique is applied to track the desired angle trajectories and a torque controller is designed to track the desired knee stiffness on both gait phases.

### 3.1 Kinematic Control

The human-exoskeleton dynamics with motor and muscle torque inputs in Eq. 1 can be expressed in terms of the kinematic control objective as

$$M_{\rho }\left(q\right)\ddot{q}+C_{\rho }\left(q,\dot{q}\right)\dot{q}+G_{\rho }\left(q\right)+P_{\rho }\left(q,\dot{q}\right)+d_{\rho }\left(t\right)=\underset{\tau_{\kappa }}{\underset{︸}{B_{\kappa }\left(q,\dot{q},t\right)u_{\kappa }\left(t\right)}}+\tau_{s},$$ (4)

∀ρ∈R , where $\tau_{\kappa },\tau_{s}\in R^{4}$ are the torque inputs generated by the kinematic and stiffness controllers, $u_{\kappa }:R_{\geq t_{0}}\to R^{4}$ is the kinematic control input, and the lumped kinematic control effectiveness $B_{\kappa }\in R_{>0}^{4\times 4}$ is a positive definite diagonal matrix, defined as

$$B_{\kappa }≜\overset{8}{\underset{e=5}{\sum }}B_{e}\left(q,\dot{q}\right)\sigma_{e}\left(t\right)+\overset{4}{\underset{m=1}{\sum }}B_{m}\left(q,\dot{q},t\right)\sigma_{m}\left(t\right),$$ (5)

where the hip motors dedicated for kinematic tracking are represented by the motor index *e* = {5, 6, 7, 8} as described in Table 1.

The measurable angular position tracking error $e_{\kappa }:R_{\geq t_{0}}\to R^{4}$ and filtered tracking error $r_{\kappa }:R_{\geq t_{0}}\to R^{4}$ are defined as.

$$\begin{array}{ll} & e_{\kappa }\left(t\right)≜q_{d}\left(t\right)-q\left(t\right), \end{array}$$ (6)

$$\begin{array}{ll} & r_{\kappa }\left(t\right)≜{\dot{e}}_{\kappa }\left(t\right)+\alpha e_{\kappa }\left(t\right), \end{array}$$ (7)

where α∈R is a selectable positive control gain and $q_{d}\left(t\right),{\dot{q}}_{d}\left(t\right),{\ddot{q}}_{d}:R_{\geq t_{0}}\to R^{4}$ are bounded desired joint trajectories. Taking the time derivative of Eq. 7, substituting for Eq. 4 and Eq. 6, and then performing algebraic manipulation yields

$${\dot{r}}_{\kappa }=\chi_{\rho }-e_{\kappa }+M_{\rho }^{-1}\left(-B_{\kappa }u_{\kappa }-\tau_{s}\right),$$ (8)

where the auxiliary signal $\chi_{\rho }:R_{\geq t_{0}}\to R^{4}$ is defined as

$$\chi_{\rho }={\ddot{q}}_{d}+\alpha {\dot{e}}_{\kappa }+e_{\kappa }+M_{\rho }^{-1}\left(C_{\rho }\dot{q}+G_{\rho }+P_{\rho }+d_{\rho }\right).$$ (9)

By using Properties 2–5, Assumption 1, Eqs 6, 7, the auxiliary signal in Eq. 9 can be upper bounded as

$$\left\|\chi_{\rho }\right\|\leq c_{1}+c_{2}\left\|z_{\kappa }\right\|+c_{3}{\left\|z_{\kappa }\right\|}^{2},\forall \rho \in R,$$ (10)

where $c_{1},c_{2},c_{3}\in R_{>0}$ are positive constants and $z_{\kappa }≜{\left[\begin{array}{cc} e_{\kappa }^{T} & r_{\kappa }^{T} \end{array}\right]}^{T}:R_{\geq t_{0}}\to R^{8}$ . Given the open-loop error system in Eq. 8, the control input $u_{\kappa }\in R^{4}$ can be designed as

$$u_{\kappa }=k_{1}r_{\kappa }+\left(k_{2}+k_{3}\left\|z_{\kappa }\right\|+k_{4}{\left\|z_{\kappa }\right\|}^{2}+k_{5}\left\|u_{s}\right\|\right)\text{sgn}\left(r_{\kappa }\right),$$ (11)

where $k_{1},k_{2},k_{3},k_{4},k_{5}\in R_{>0}$ are selectable positive gains, and *u*_*s*_ is a subsequently designed stiffness control input. The kinematic control input in Eq. 11 includes a feedback term and robust control terms to reject the disturbance, and compensate for the state-dependent uncertain terms in Eq. 9, and compensate for the stiffness input cross-term. The closed-loop error system can be obtained by substituting Eq. 11 into the open-loop error system Eq. 8 as

$${\dot{r}}_{\kappa }=\chi_{\rho }-M_{\rho }^{-1}\tau_{s}-e_{\kappa }-M_{\rho }^{-1}B_{\kappa }\left(k_{1}r_{\kappa }+\left(k_{2}+k_{3}\left\|z_{\kappa }\right\|+k_{4}{\left\|z_{\kappa }\right\|}^{2}+k_{5}\left\|u_{s}\right\|\right)\text{sgn}\left(r_{\kappa }\right)\right).$$ (12)

Remark 1 *To implement* Eq. 11 *, u*_*s*_ *is initialized at zero* (*i.e.,* [0,0]^*T*^)*, such that* $\left\|u_{s}\right\|$ *is bounded at t* = *t*_0_ *.*

### 3.2 Stiffness Control

The stiffness control objective is to track a desired torque for the knee joints. Hence, the knee-shank dynamics in Eq. 1 can expressed as

$$M_{\rho k}\left(q_{k}\right){\ddot{q}}_{k}+C_{\rho k}\left(q_{k},{\dot{q}}_{k}\right){\dot{q}}_{k}+G_{\rho k}\left(q_{k}\right)+P_{\rho k}\left(q_{k},{\dot{q}}_{k}\right)+d_{\rho k}\left(t\right)=\tau_{\kappa k}\left(t\right)+\underset{\tau_{sk}}{\underset{︸}{B_{s}\left(q,\dot{q}\right)u_{s}\left(t\right)}},$$ (13)

where the subscript *k* refers to the knee-joint dynamics, $q_{k},{\dot{q}}_{k}\in R^{2}$ are the knee joint angles and velocities, respectively. The terms $M_{\rho k}\in R_{>0}^{2\times 2}$ , $C_{\rho k}\in R^{2\times 2}$ , and $G_{\rho k}\in R^{2}$ denote the inertia, centripetal-Coriolis, and gravitational effects, respectively; $P_{\rho k}\in R^{2}$ , $\tau_{\kappa k}\in R^{2}$ denote damping and viscoelastic effects, and torque applied about the knee joint by the kinematic controller, respectively; $d_{\rho k}\in R^{2}$ denotes unmodeled terms and disturbances acting about the knee joints (e.g., interaction forces induced by the hip joints). The stiffness control input is denoted by $u_{s}:R_{\geq t_{0}}\to R^{2}$ and the lumped stiffness control effectiveness $B_{s}\in R_{>0}^{2\times 2}$ is a positive definite diagonal matrix, defined as

$$B_{s}≜\overset{4}{\underset{e=1}{\sum }}B_{e}\left(q,\dot{q}\right)\sigma_{e}\left(t\right),$$ (14)

where the effectiveness dimension has been reduced from 4 × 4 to 2 ×2 since the stiffness control objective is developed only for the knee joints. Hence, the torque due to the stiffness controller *τ*_*s*_ about the hip joints is zero (i.e., $\tau_{s}={\left[\tau_{sk}^{T},0,0\right]}^{T}$ ). The knee joint torque inputs generated by the kinematic and stiffness controllers are denoted as $\tau_{\kappa k},\tau_{sk}\in R^{2}$ , respectively.

To generate the desired torque, a stiffness model is designed as

$$\tau_{d}\left(t\right)=K\left(t\right)\left(q_{dk}-q_{k}\right),$$ (15)

where $K\left(t\right):R_{\geq t_{0}}\to R_{>0}^{2\times 2}$ is a selectable positive definite diagonal matrix representing virtual knee-joint springs, and $\tau_{d}\in R^{2}$ denotes the generated desired knee torque trajectories. The desired spring matrix is designed using Fourier series with periodic, continuous and differentiable properties, such that $\bar{K}{\left\|\zeta \right\|}^{2}\leq \zeta^{T}K\left(t\right)\zeta \leq \underline{K}{\left\|\zeta \right\|}^{2}$ , $\forall \zeta \in R^{2}$ , where $\bar{K}$ and $\underline{K}$ are known positive constants denoting the upper and lower bounds of *K*, respectively.

An integral-like torque tracking error $e_{s}:R_{\geq t_{0}}\to R^{2}$ is defined as

$$e_{s}\left(t\right)≜\int_{t_{0}}^{t}\left(\tau_{d}\left(\varphi \right)-\tau_{k}\left(\varphi \right)\right)d\varphi ,$$ (16)

where *τ*_*k*_ ≜ *τ*_*κk*_ + *τ*_*sk*_ is the measurable torque applied about the knee joints. Taking the derivative of Eq. 16, setting the initial conditions to zero, and substituting the measurable torque inputs from the right-hand side in Eqs 13, 15 yields

$${\dot{e}}_{s}\left(t\right)=K\left(t\right)e_{\kappa k}-\tau_{\kappa k}-B_{s}u_{s},$$ (17)

where $e_{\kappa k},{\dot{e}}_{\kappa k}\in R^{2}$ are the knee joint position and velocity tracking errors defined as *e*_*κk*_ ≜ *q*_*dk*_ − *q*_*k*_, ${\dot{e}}_{\kappa k}≜{\dot{q}}_{dk}-{\dot{q}}_{k}$ . The stiffness control input $u_{s}\in R^{2}$ is designed as

$$u_{s}\left(t\right)=k_{6}e_{s}+\left(k_{7}e_{\kappa k}+k_{8}\left\|u_{\kappa k}\right\|\right)\text{sgn}\left(e_{s}\right),$$ (18)

where $k_{6},k_{7},k_{8}\in R_{>0}$ are selectable positive control gains and $u_{\kappa k}\in R^{2}$ is the knee joint kinematic controller input. The closed-loop stiffness error system is obtained by substituting Eq. 18 into Eq. 17 to yield

$${\dot{e}}_{s}=K\left(t\right)e_{\kappa k}-\tau_{\kappa k}-B_{s}\left(k_{6}e_{s}+\left(k_{7}e_{\kappa k}+k_{8}\left\|u_{\kappa k}\right\|\right)\text{sgn}\left(e_{s}\right)\right).$$ (19)

### 3.3 Actuator Control Inputs

The kinematic and stiffness control tracking objectives combine muscle and motor inputs. Hence, the relationship between the implementable control inputs *u*_*e*_ and *u*_*m*_ (depicted in Figure 3) and the designed *u*_*κ*_ and *u*_*s*_ can be defined as

$$\begin{array}{ll} & u_{e}=k_{e}\left(D_{e}u_{\kappa }+D_{s}u_{s}\right), \end{array}$$ (20)

$$\begin{array}{ll} & u_{m}=k_{m}D_{m}u_{\kappa }, \end{array}$$ (21)

where $D_{e}=\mathrm{diag}\left(\left[\begin{array}{cccc} 0 & 0 & 1 & 1 \end{array}\right]\right)$ , $D_{s}={\left[\begin{array}{cccc} 1 & 0 & 0 & 0\\ 0 & 1 & 0 & 0 \end{array}\right]}^{T}$ , and $D_{m}=\mathrm{diag}\left(\left[\begin{array}{cccc} 1 & 1 & 0 & 0 \end{array}\right]\right)$ are control allocation matrices, diag denotes diagonal matrices, and $k_{e},k_{m}\in R_{>0}$ , ∀m∈M,∀e∈E are selectable positive control gains for the electric motors and muscle groups, respectively.

## 4 Stability Analysis

The stability of the kinematic and stiffness controllers that activate the electric motors and muscles can be examined independently through the following two theorems. Theorem 1 shows that given the closed-loop kinematic error system in Eq. 12, the joint kinematic controller in Eq. 11 achieves exponential tracking. Theorem 2 shows that given the closed-loop stiffness error system in Eq. 19, the torque controller in Eq. 18 achieves exponential tracking. All the control inputs and error signals are shown to be bounded.

**Theorem 1** *Given the closed-loop error system in* Eq. 12 *, the controller in* Eq. 11 *ensures exponential tracking in the sense that*

$$\|z_{\kappa }\|\leq \sqrt{\frac{\lambda_{\bar{\kappa }}}{\lambda_{\underline{\kappa }}}}\|z_{\kappa }\left(t_{0}\right)\|\exp\left(-\frac{\psi_{\kappa }}{2}\left(t-t_{0}\right)\right),$$ (22)

provided the following sufficient gain conditions are satisfied

$$k_{2}\geq \frac{c_{1}c_{M}}{{\underline{B}}_{\kappa }},k_{3}\geq \frac{c_{2}c_{M}}{{\underline{B}}_{\kappa }},k_{4}\geq \frac{c_{3}c_{M}}{{\underline{B}}_{\kappa }},k_{5}\geq \frac{c_{M}{\bar{B}}_{s}}{c_{m}{\underline{B}}_{\kappa }}.$$ (23)

Proof. Let $V_{\kappa }:R^{4}\times R^{4}\times R_{\geq t_{0}}\to R$ be a nonnegative, continuously differentiable function defined as

$$V_{\kappa }=\frac{1}{2}{\;e}_{\kappa }^{T}e_{\kappa }+\frac{1}{2}r_{\kappa }^{T}r_{\kappa },$$ (24)

which satisfies the following inequalities

$$\lambda_{\underline{\kappa }}{\left\|z_{\kappa }\right\|}^{2}\leq V_{\kappa }\left(z_{\kappa },t\right)\leq \lambda_{\bar{\kappa }}{\left\|z_{\kappa }\right\|}^{2},$$ (25)

where $\lambda_{\underline{\kappa }},\lambda_{\bar{\kappa }}\in R_{>0}$ are known positive bounding constants. The control input in Eq. 11 has the discontinuous signum function (i.e., sliding-mode), and the torque inputs in Eqs 2, 3 have input switching effects; hence, the system’s trajectories cannot be solved in a classical sense. Let *z*_*κ*_(*t*) be a Filippov solution to the differential inclusion ${\dot{z}}_{\kappa }\in K\left[h_{\kappa }\right]\left(z_{\kappa }\right)$ , where K[⋅] is defined as Paden and Sastry (1987) and *h*_*κ*_ is defined using Eqs 7, 12 as $h_{\kappa }≜\left[\begin{array}{cc} h_{1} & h_{2} \end{array}\right]$ , where *h*_1_ ≜ *r*_*κ*_ − *αe*_*κ*_ and $h_{2}≜\chi_{\rho }-M_{\rho }^{-1}\tau_{s}-e_{\kappa }-M_{\rho }^{-1}K\left[B_{\kappa }\right]\left(k_{1}r_{\kappa }+\left(k_{2}+k_{3}\left\|z_{\kappa }\right\|+k_{4}{\left\|z_{\kappa }\right\|}^{2}+k_{5}\left\|u_{s}\right\|\right)\;K\left[\text{sgn}\left(r_{\kappa }\right)\right]\right)$ . Hence, the time derivative of Eq. 24 exists almost everywhere (a.e.), i.e., for almost all time. Based on (Fischer et al., 2013, Lemma 1), the time derivative of Eq. 24, ${\dot{V}}_{\kappa }\left(z_{\kappa }\left(t\right),t\right)\overset{a.e.}{\in }{\dot{\tilde{V}}}_{\kappa }\left(z_{\kappa }\left(t\right),t\right)$ , where ${\dot{\tilde{V}}}_{\kappa }$ is the generalized time derivative of Eq. 24 along the Filippov trajectories of ${\dot{z}}_{\kappa }=h_{\kappa }\left(z_{\kappa }\right)$ and is defined as in Fischer et al. (2013) as ${\dot{\tilde{V}}}_{\kappa }≜{⋂}_{\xi \in \partial V_{\kappa }}\xi^{T}K{\left[\begin{array}{ccc} {\dot{e}}_{\kappa } & {\dot{r}}_{\kappa } & 1 \end{array}\right]}^{T}\left(e_{\kappa },r_{\kappa },t\right)$ . Since *V*_*κ*_(*z*_*κ*_, *t*) is continuously differentiable in *z*_*κ*_, *∂V*_*κ*_ = {∇*V*_*κ*_}, thus ${\dot{\tilde{V}}}_{\kappa }\overset{\text{a.e.}}{\subset }\left[\begin{array}{cc} e_{\kappa } & r_{\kappa } \end{array}\right]K{\left[\begin{array}{cc} {\dot{e}}_{\kappa } & {\dot{r}}_{\kappa } \end{array}\right]}^{T}$ . Therefore, after taking the time derivative, the generalized time derivative of Eq. 24 can be expressed as ${\dot{\tilde{V}}}_{\kappa }\overset{a.e.}{\subset }e_{\kappa }^{T}{\dot{e}}_{\kappa }+r_{\kappa }^{T}{\dot{r}}_{\kappa }$ . After substituting Eqs 6, 7, 12, the generalized time derivative of Eq. 24 can be expressed as

$$\begin{array}{l} {\dot{\tilde{V}}}_{\kappa }\overset{a.e.}{\subset }-e_{\kappa }^{T}\alpha e_{\kappa }+r_{\kappa }^{T}\chi_{\rho }-r_{\kappa }^{T}M_{\rho }^{-1}\tau_{s}-r_{\kappa }^{T}M_{\rho }^{-1}K\left[B_{\kappa }\right]\left(k_{1}r_{\kappa }+\left(k_{2}+k_{3}\left\|z_{\kappa }\right\|+k_{4}{\left\|z_{\kappa }\right\|}^{2}+k_{5}\left\|u_{s}\right\|\right)K\left[\text{sgn}\left(r_{\kappa }\right)\right]\right). \end{array}$$ (26)

The generalized time derivative of (24) can be upper bounded using Property 6 as

$$\begin{array}{l} {\dot{\tilde{V}}}_{\kappa }\overset{a.e.}{\leq }-\alpha {\left\|e_{\kappa }\right\|}^{2}-\frac{{\underline{B}}_{\kappa }}{c_{M}}k_{1}{\left\|r_{\kappa }\right\|}^{2}+\left(c_{1}-k_{2}\frac{{\underline{B}}_{\kappa }}{c_{M}}\right)\left\|r_{\kappa }\right\|+\left(c_{2}-k_{3}\frac{{\underline{B}}_{\kappa }}{c_{M}}\right)\left\|r_{\kappa }\right\|\left\|z_{\kappa }\right\|\\ +\left(c_{3}-k_{4}\frac{{\underline{B}}_{\kappa }}{c_{M}}\right)\left\|r_{\kappa }\right\|{\left\|z_{\kappa }\right\|}^{2}+\left(\frac{{\bar{B}}_{s}}{c_{m}}-k_{5}\frac{{\underline{B}}_{\kappa }}{c_{M}}\right)\left\|r_{\kappa }\right\|\left\|u_{s}\right\|. \end{array}$$ (27)

Provided the gain conditions in Eq. 23 are satisfied, the inequality in Eq. 27 can be further upper bounded as

$${\dot{\tilde{V}}}_{\kappa }\overset{a.e.}{\leq }-W\left(z_{\kappa }\right),$$ (28)

where $W≜\alpha {\left\|e_{\kappa }\right\|}^{2}+\frac{{\underline{B}}_{\kappa }}{c_{M}}k_{1}{\left\|r_{\kappa }\right\|}^{2}$ is a positive definite function; hence, Eq. 28 satisfies the conditions in Liberzon (2003) to guarantee that Eq. 24 is a common Lyapunov function across subsystems *ρ* = {1, 2} (i.e., stance and swing phases of walking). The upper bound in Eq. 25 can be substituted into the previous expression to yield

$${\dot{\tilde{V}}}_{\kappa }\overset{a.e.}{\leq }-\psi_{\kappa }{\tilde{V}}_{\kappa },$$ (29)

where $\psi_{\kappa }≜\frac{1}{\lambda_{\bar{\kappa }}}\min\left(\alpha ,\frac{{\underline{B}}_{\kappa }}{c_{M}}k_{1}\right)$ . Leveraging Eqs 25, 29, the result in Eq. 22 can be obtained. Using Eqs 24, 29, $V_{\kappa }\in L_{\infty }$ , hence, $e_{\kappa },r_{\kappa }\in L_{\infty }$ , which implies that $z_{\kappa }\in L_{\infty }$ , and thus $q,\dot{q}\in L_{\infty }$ .

**Theorem 2** Given the closed-loop error system in Eq. 19, the controller in Eq. 18 ensures exponential tracking in the sense that

$$\|e_{s}\|\leq \|e_{s}\left(t_{0}\right)\|\exp\left(-\frac{\psi_{s}}{2}\left(t-t_{0}\right)\right),$$ (30)

provided the following sufficient gain conditions are satisfied

$$k_{7}\geq \frac{\bar{K}}{{\underline{B}}_{s}},k_{8}\geq \frac{{\bar{B}}_{\kappa }}{{\underline{B}}_{s}}.$$ (31)

Proof. Let $V_{s}:R^{2}\times R_{\geq t_{0}}\to R$ be a nonnegative, continuously differentiable function defined as

$$V_{s}=\frac{1}{2}{\;e}_{s}^{T}e_{s}.$$ (32)

Let *e*_*s*_(*t*) be a Filippov solution to the differential inclusion ${\dot{e}}_{s}\in K\left[h\right]\left(z_{q}\right)$ , where K[⋅] is defined as Paden and Sastry (1987) and *h* ≜ *h*_3_ is defined by using Eq. 19 as $h_{3}≜K\left(t\right)e_{\kappa k}-\tau_{\kappa k}-K\left[B_{s}\right]\left(k_{6}e_{s}+\left(k_{7}e_{\kappa k}+k_{8}\left\|u_{\kappa k}\right\|\right)K\left[\text{sgn}\left(e_{s}\right)\right]\right)$ . The control input in Eq. 18 includes the discontinuous signum function and the closed-loop error system in Eq. 19 has the lumped switched stiffness control effectiveness. Hence, the time derivative of Eq. 32 exists almost everywhere (a.e.), i.e., for almost all time. After substituting for Eq. 19 and using similar arguments as in the proof of Theorem 1, the generalized time derivative of Eq. 32 can be expressed as

$${\dot{\tilde{V}}}_{s}\overset{a.e.}{\subset }e_{s}^{T}\left(K\left(t\right)e_{\kappa k}-\tau_{\kappa k}-K\left[B_{s}\right]\left(k_{6}e_{s}+\left(k_{7}e_{\kappa k}+k_{8}\left\|u_{\kappa k}\right\|\right)K\left[\text{sgn}\left(e_{s}\right)\right]\right)\right).$$ (33)

An upper bound for the previous expression can be obtained by using Property 7 and substituting the upper bound of *K*(*t*) to yield

$${\dot{\tilde{V}}}_{s}\overset{a.e.}{\leq }-{\underline{B}}_{s}k_{6}{\left\|e_{s}\right\|}^{2}+\left\|e_{s}\right\|\left\|e_{\kappa k}\right\|\left(\bar{K}-k_{7}{\underline{B}}_{s}\right)+\left\|e_{s}\right\|\left\|u_{\kappa k}\right\|\left({\bar{B}}_{\kappa }-k_{8}{\underline{B}}_{s}\right).$$ (34)

Provided the gain conditions in Eq. 31 are satisfied, the inequality in Eq. 34 can be further upper bounded as

$${\dot{\tilde{V}}}_{s}\overset{a.e.}{\leq }-\psi_{s}{\tilde{V}}_{s},$$ (35)

where $\psi_{s}≜{\underline{B}}_{s}k_{6}$ . Using Eqs 32, 35, $V_{s}\in L_{\infty }$ , hence, $e_{s}\in L_{\infty }$ . Given the fact that $e_{\kappa }\in L_{\infty }$ from Theorem 1, which implies that $\tau_{d}\in L_{\infty }$ in Eq. 15, then, $\tau_{k}\in L_{\infty }$ in Eq. 16. Based on *τ*_*k*_ ≜ *τ*_*κk*_ + *τ*_*sk*_, leveraging Remark 1, and substituting Eq. 11 in *τ*_*κk*_, it can be concluded that $u_{s}\in L_{\infty }$ . Thus, from Eq. 11 $u_{\kappa }\in L_{\infty }$ , which further implies that $u_{m},u_{e}\in L_{\infty }$ from Eqs 20, 21.

## 5 Experiment Results

Experiments are provided to demonstrate the performance of the kinematic and stiffness controllers developed in Eqs 11, 18 to control the knee and hip joints. The control inputs are commanded as stimulation intensities (i.e., pulse width control) to activate the quadriceps and hamstring muscle groups and as currents to the electric motors. Three able-bodied individuals (two males aged 29 years and one female aged 29 years) participated in the exoskeleton protocol at Syracuse University. Written informed consent was obtained from each participant, as approved by the Institutional Review Board at Syracuse University. The participants were instructed to avoid voluntarily contributing to the treadmill walking task. To mitigate the influence of the ankle joint for propulsion, an orthotic boot is used to mechanically lock the ankle and provide foot cushion. The individuals could not see the walking performance plots during the experiments.

**FIGURE 5:**
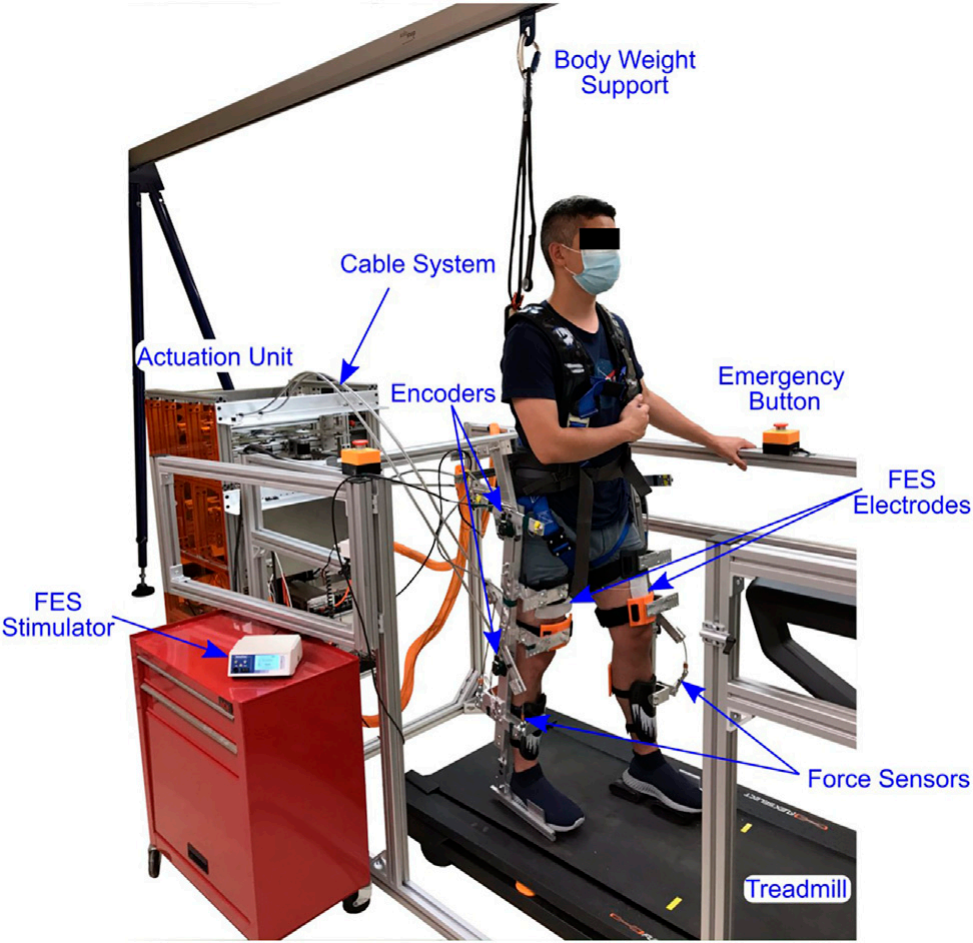
The exoskeleton testbed used for treadmill walking. The exoskeleton uses cables to apply torque at each joint using electric motors installed in the actuation unit. Surface FES is applied on the quadriceps and hamstrings muscle groups. Additional components of the walking system are labeled in the image.

Testing were performed using a customized exoskeleton designed for fitting different body sizes and maintaining alignment with the user’s joints. Figure 5 illustrates the exoskeleton testbed. The actuator unit includes brushless 24 VDC electric motors (Maxon) to adjust the torque applied by the cable-driven mechanisms. Optical encoders (US Digital) were mounted at each joint to measure the joint angle and load-cells (OMEGA) were installed in series with the cables to measure cable tension. The controllers were implemented on a desktop computer (Windows 10 OS) running a real-time target (QUARC 2.6, Quanser) *via* MATLAB/Simulink 2018a (MathWorks Inc) with a sample rate of 1 kHz. The Quanser QPIDe DAQ board was used to read the encoders and cable tensions, and control the servo motor drivers (Maxon) operating in current-controlled mode. The Quanser Q8 USB board was used to read the encoders mounted on motors. A current-controlled stimulator (RehaStim, Hasomed GmbH) delivered biphasic, symmetric, rectangular pulses to the participant’s quadriceps and hamstring muscle groups. Self-adhesive PALS^®^ electrodes (3″ by 5″)^1^ were placed on each muscle group in both legs. The stimulation current amplitude and stimulation frequency were fixed at 80 mA and 60 Hz, respectively. A treadmill (Nordic Track) equipped with an encoder (US Digital) to measure the belt’s angular displacement was used for walking at two constant speeds: 0.5 and 0.8 mph. The speed of the treadmill was closed-loop controlled using a sliding-mode controller and implemented in a motor driver in current-control mode (Advanced Motion Controls)^2^. As safety measures, the participant had access to an emergency stop button and software stop conditions were implemented to limit the amount of motor currents to comply with the hardware limits and prevent large current transients from being applied to the participant, and muscle stimulation intensities to prevent uncomfortable stimulation intensities. Mechanical stops were designed to avoid moving the legs through unsafe joint angles, and the participants wear a safety harness connected to a portable track system to prevent falling without restricting the motion.

A walking pretrial was performed wearing the exoskeleton in passive mode (i.e., the exoskeleton did not provide assistance to the participant) to record walking data for each participant to generate the smooth desired kinematic trajectories $q_{d},{\dot{q}}_{d}$ . The desired stiffness values $K\left(t\right)≜\mathrm{diag}\left(\left[\begin{array}{cc} K_{R} & K_{L} \end{array}\right]\right)$ were designed using Fourier series as $K_{R}\left(t\right)=K_{L}\left(t+1\right)=\frac{1}{2}a_{0}+\sum_{n=1}^{30}b_{n}\sin\left(n\pi t\right)$ , where $a_{0}=K_{1}+K_{2},b_{n}=\left(\frac{1}{n\pi }\right)\left(K_{2}-K_{1}+K_{1}{\left(-1\right)}^{n}-k_{2}{\left(-1\right)}^{n}\right)$ with *K*_1_ = 10, *K*_2_ = 4. The control gains were tuned to achieve satisfactory tracking performance during preliminary testing following the guidelines described in the Appendix. The control gains introduced in Eqs 11, 18 were selected as follows: *k*_1_ = 0.4, *k*_2_ = 0.35, *k*_3_ = 0.002, *k*_4_ = 0.0001, *k*_5_ = 0.05, *α* = 20, *k*_6_ = 0.05, *k*_7_ = 6.5, *k*_8_ = 0.05. The selectable positive control gains in Eqs 20, 21 are *k*_*e*_ = 0.8 and *k*_*m*_ ∈ [8, 12]. The joint torque *τ*_*k*_ were computed in real-time based on the force measurement from the load cells multiplied by the computed moment arm, which is a function of joint angles. The treadmill walking experiments have a duration of 3 min.

**FIGURE 6:**
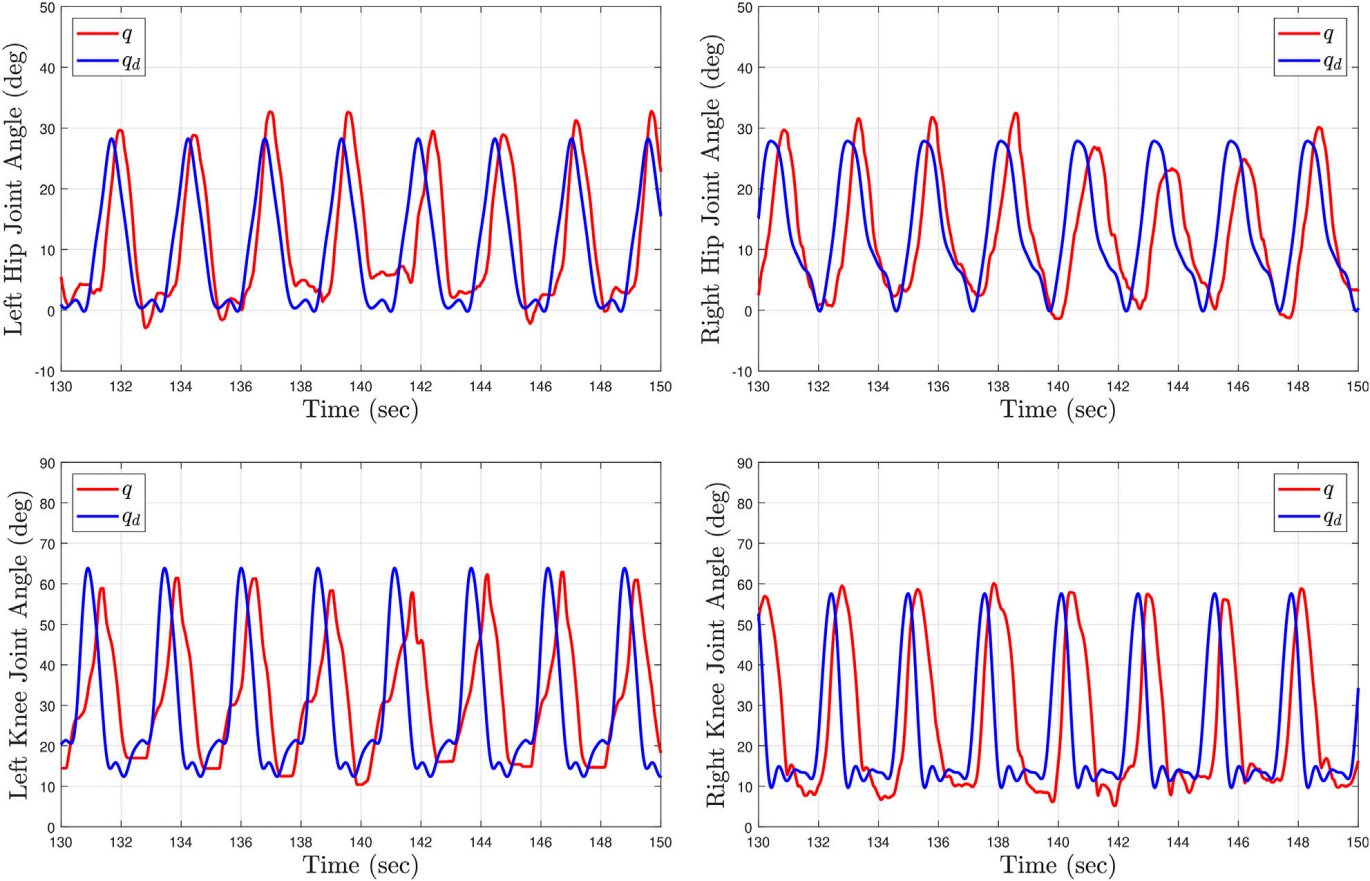
Kinematic tracking performance for Subject 2 (S2) after 2 minutes of treadmill walking at high speed (0.8 mph). The top plots depict the left and right hip joint kinematics. The bottom plots depict the left and right knee joint kinematics. The blue curves illustrate the desired kinematic trajectories and the red curves show the actual joint angles.

**FIGURE 7:**
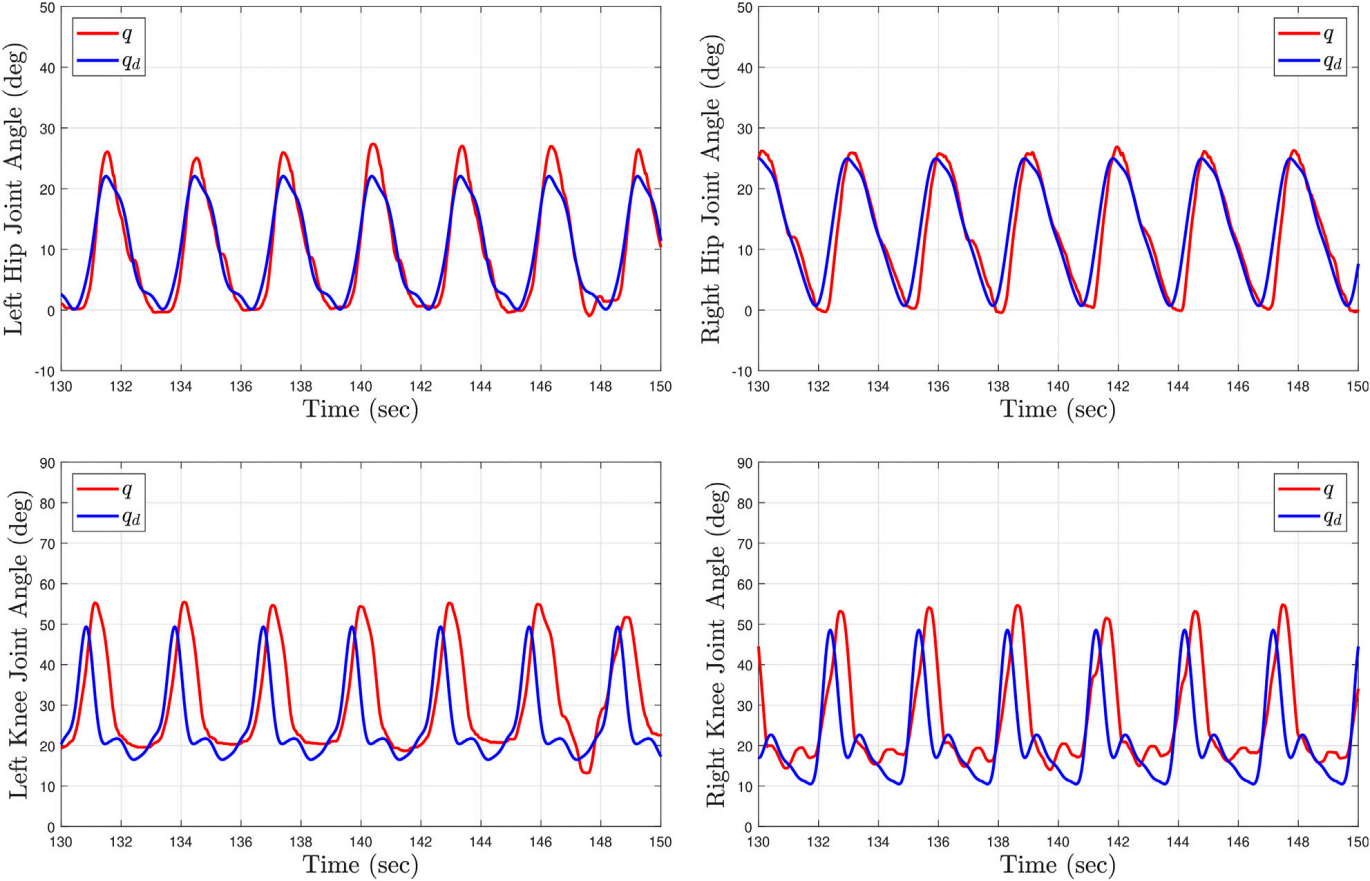
Kinematic tracking performance for Subject 1 (S1) after 2 minutes of treadmill walking at low speed (0.5 mph). The top plots depict the left and right hip joint kinematics. The bottom plots depict the left and right knee joint kinematics. The blue curves illustrate the desired kinematic trajectories and the red curves show the actual joint angles.

**FIGURE 8:**
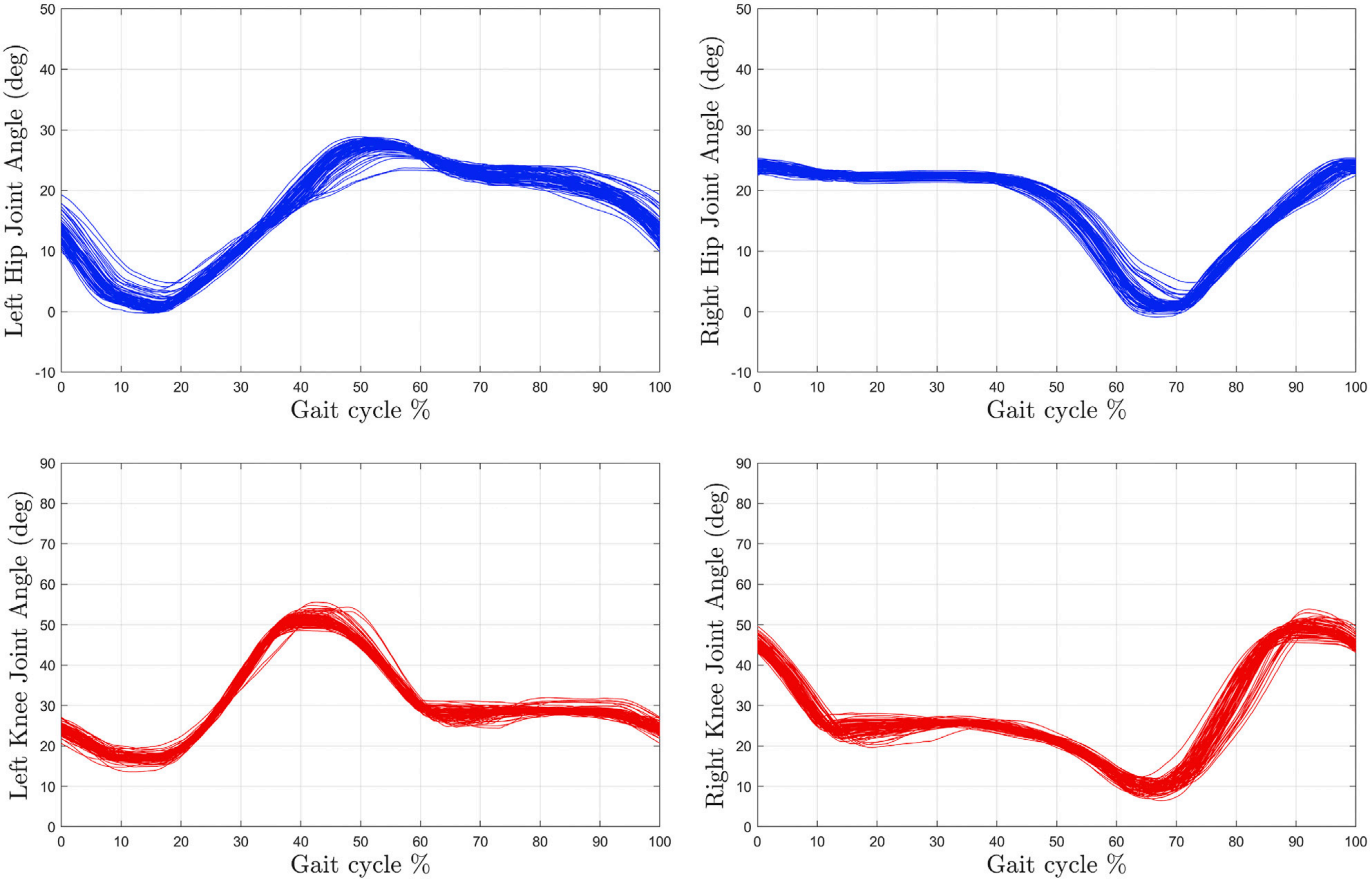
The kinematic joint trajectories corresponding to each gait cycle during the treadmill walking experiment at high speed (0.8 mph) for Subject 1 (S1).

Table 2 summarizes the root-mean-squared (RMS) and average of the kinematic and stiffness tracking errors for all subjects with the two tested gait speeds. The experimental results were analyzed after the sixth gait cycle from the point at which the treadmill reached the desired steady-state gait speed. During the first five gait cycles the participants began stepping on the treadmill to smoothly reach the steady state constant walking speed. The kinematic tracking performance for two participants at different gait speeds is illustrated in Figures 6, 7, where the desired joint trajectories are depicted in blue and the actual joint angles are depicted in red. The kinematic joint trajectories corresponding to each gait cycle during a complete treadmill walking experiment are depicted as a function of gait cycle percentage in Figure 8.

**TABLE 2:** Tracking results for each participant at high (0.8 mph) and low (0.5 mph) treadmill walking speeds^a^: RMS kinematic tracking error (moving window in seconds to complete a gait cycle)^b^, average of kinematic tracking error $\bar{e_{\kappa }}$ , and average of stiffness tracking error ${\bar{e_{s}}}^{c}$ .

Subject-speed	Leg	RMS kinematic error (deg)		$\bar{e_{\kappa }}$ **(deg)**		$\bar{e_{s}}$ **(Nms)**
		Knee	Hip	Knee	Hip	Knee
S1-High	R	2.6 ± 1.8	2.1 ± 1.2	−0.1 ± 1.3	−0.5 ± 0.8	37.9 ± 6.0
	L	5.4 ± 3.2	2.3 ± 1.5	−5.2 ± 1.9	−1.3 ± 0.7	37.3 ± 4.7
S1-Low	R	3.5 ± 2.0	1.1 ± 1.0	−3.1 ± 1.9	−0.4 ± 1.2	40.5 ± 21.4
	L	3.7 ± 2.0	2.4 ± 2.0	−3.6 ± 1.3	−2.2 ± 1.5	67.7 ± 27.4
S2-High	R	3.0 ± 2.2	1.4 ± 1.0	0.0 ± 2.6	1.1 ± 0.8	57.3 ± 21.2
	L	2.6 ± 1.8	1.2 ± 0.9	−1.4 ± 1.8	−0.9 ± 0.7	90.9 ± 31.0
S2-Low	R	5.0 ± 3.2	2.4 ± 1.5	3.9 ± 4.9	−0.1 ± 2.5	23.6 ± 9.7
	L	8.5 ± 4.0	2.4 ± 1.5	8.5 ± 1.9	0.9 ± 1.2	−40.4 ± 12.7
S3-High	R	1.5 ± 0.9	4.7 ± 0.9	−1.0 ± 1.2	−4.7 ± 1.9	16.9 ± 6.0
	L	3.8 ± 2.1	3.8 ± 1.9	3.7 ± 3.5	−3.7 ± 3.4	14.1 ± 4.9
S3-Low	R	8.2 ± 1.5	3.9 ± 1.5	−8.3 ± 1.4	−4.0 ± 1.9	30.0 ± 11.4
	L	2.5 ± 1.8	4.4 ± 3.3	−1.7 ± 2.7	−4.1 ± 3.9	39.6 ± 15.6
Mean (S1–S3)		4.2	2.7	−0.7	−1.7	34.6
STD(S1–S3)^d^		2.2	1.5	2.2	1.7	14.3

a Reported as mean ± standard deviation (STD).

b Moving window is selected for each participant based on his/her step length. For the three participants, the moving window is selected within the range of 1.7–2.3 s for high speed walking and 2.7–3.6 s for low speed walking.

c Averages evaluated over the gait cycle. The gait cycle starts with heel strike in the right leg.

d Reports the mean over the standard deviations.

**FIGURE 9:**
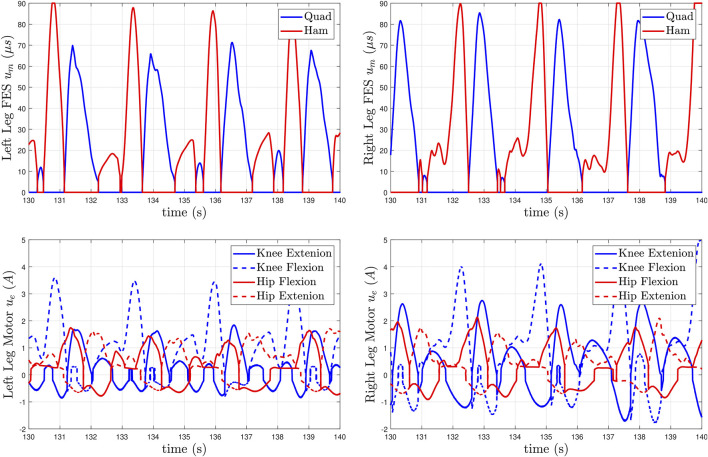
Distribution of the muscle and motor control inputs for Subject 2 (S2) during the high speed walking trial (0.8 mph). The top plots depict the pulse widths generated by *u*_*m*_ and applied to the quadriceps and hamstrings muscle groups for the left and right legs after 2 minutes of treadmill walking. The bottom plots show the motor currents generated by *u*_*e*_ and applied to the motors that actuate the knee and hip joints of the left and right legs.

The control inputs are presented in Figure 9, the quadriceps and hamstrings muscle stimulation inputs *u*_*m*_ for both legs are displayed at the top, whereas the electric motor input commands *u*_*e*_ are depicted at the bottom. The muscle input switching is observed through the activation of hams and quads to achieve flexion and extension, respectively. Similarly, the motor commands are switching between the upper layer command *u*_*m*_ and the lower layer synchronization control commands *u*_*fl*_ and *u*_*ex*_ as depicted in Figure 3.

**FIGURE 10:**
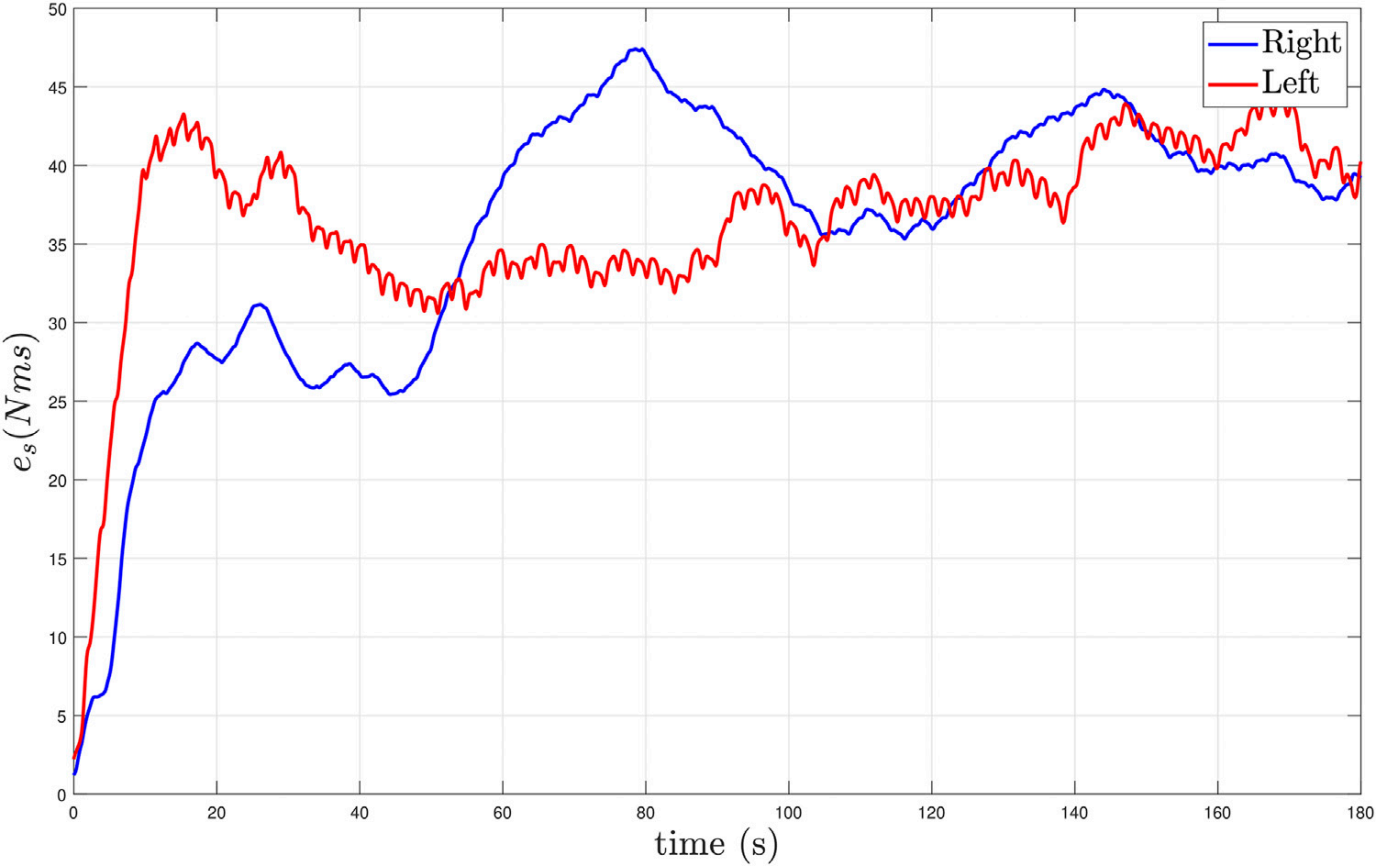
Stiffness tracking performance during treadmill walking at high gait speed (0.8 mph) for Subject 1 (S1). The stiffness tracking performance is depicted in blue and red for the right and left knee joints, respectively. The data is presented with a moving time interval window of 1.99 s, which is the time in seconds to complete a gait cycle.

**FIGURE 11:**
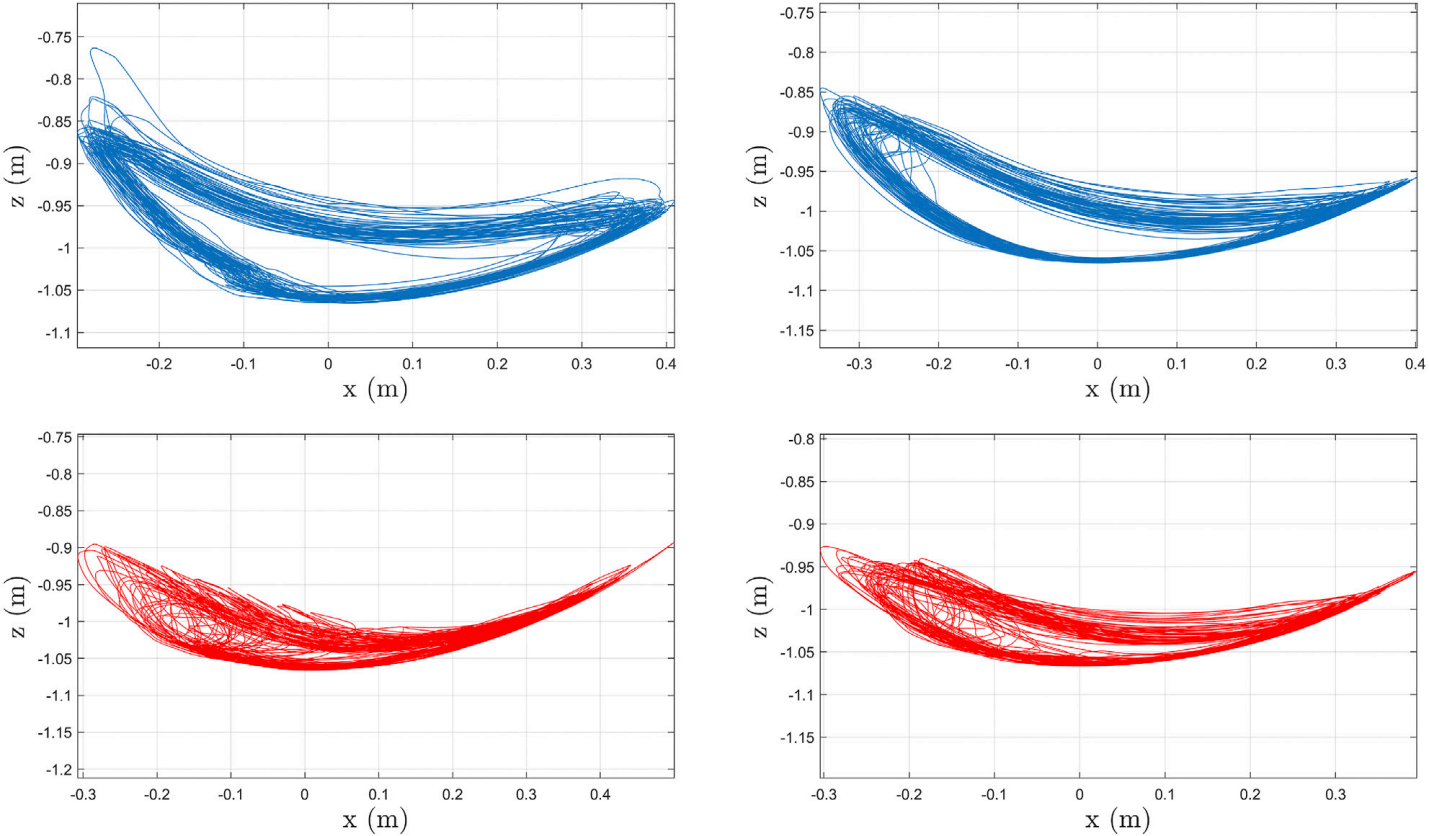
Foot trajectories in the sagittal plane (i.e., the foot path in the x-z plane) at high **(top)** and slow **(bottom)** speeds for Subject 2 (S2). The plots in the left column correspond to the left leg and plots in the right column correspond to the right leg. The trajectories are computed using collected joint angles, where the origin corresponds to the trunk position.

The stiffness tracking errors for the left and right knee joints are presented in Figure 10. The stiffness errors in Figure 10 are quantified using a moving time interval window of 1.99 s, which is selected based on the walking speed and step length of the participant. Both integral stiffness error signals remain bounded during the experiment. Figure 11 depicts the computed foot trajectories in the sagittal plane for the two gait speeds, which further illustrates the influence of the developed controllers and gait speed on the participant’s walking pattern.

## 6 Discussion

The experimental results demonstrate the feasibility of the controllers developed in Eqs 11, 18 to activate lower-limb muscles *via* FES and provide torque assistance about the knee and hip joints. The designed controllers exploited kinematic and torque feedback to achieve treadmill walking at a constant speed. By adjusting the tuning of the stiffness controller, the exoskeleton provides higher or lower leg compliance, which directly influences the joint kinematics. In addition, adjusting the tuning of the muscle kinematic controllers can customize the stimulation intensities applied to the quadriceps and hamstrings muscle groups. Therefore, coupling kinematic and stiffness controllers for FES and electric motors can influence each individual’s gait kinematics and foot trajectories across different walking speeds as illustrated in Figure 8 and Figure 11. As depicted in Figure 8, the developed controllers achieved repeatable and consistent kinematic joint trajectories as a function of the gait cycle for participant S1. Moreover, consistent joint kinematic patterns were obtained across all participants for both walking speeds, which are described with a group average of the joint angle standard deviations: ± 5.85° for the right knee, ± 5.03° for the right hip, ± 8.57° for the left knee, and ±5.88° for the left hip. Similarly, Figure 11 denotes the foot trajectories in the sagittal plane, which are computed based on joint kinematics. The foot path is another indirect metric of gait consistency, which could be further used to quantify human performance or design alternate control methods. The integration of FES and cable-driven exoskeletons holds the potential to customize the human interaction to restore or improve function in individuals with movement disorders by achieving repetitive and coordinated walking.

The results in this study align qualitatively with previously published results for exoskeletons that include FES to activate lower-limb muscles. However, the differences in the control designs and experimental test beds pose challenges to directly compare the obtained results in this study with previous studies on hybrid exoskeletons. In Alibeji et al. (2018a,b) a hybrid neuroprosthesis (i.e., a powered exoskeleton with surface FES) was tested in one able-bodied individual and one participant with SCI. The performance of the designed muscle and motor controllers was demonstrated during overground walking assisted by a walker. In Ha et al. (2016), a cooperative control approach was used to iteratively compute the muscle stimulations during walking assisted by an exoskeleton in individuals with SCI. Despite the advances in hybrid approaches to enable assisted walking in individuals with paralysis for function restoration, technical innovations are still needed to achieve speeds and distances for walking in the community Chang et al. (2020).

The joint tracking performance is influenced by the implementation of the controllers and the unique characteristic of each individual. The hip joint kinematic tracking objective was achieved by the electric motors. Improved hip kinematic performance was obtained compared to the knee joint kinematic tracking as depicted in Figure 7 for S2 and reported in Table 2 across both treadmill walking speeds. Alternatively, the knee joint kinematic tracking objective was achieved by the activation of muscles via FES. Despite achieving the desired range of motion, the knee joint tracking performance was negatively influenced by the muscle activation input delay across all participants (as discussed in more detail in the subsequent paragraph). The electric motors controlled the stiffness objective in the knee joint to adjust the cable tensions and provide a smooth interaction for the shank throughout the gait cycle. An alternate approach could be for the muscles and electric motors to cooperate to achieve improved knee joint tracking performance. However, the cooperative control of muscles and motors has to be carefully selected to avoid the exoskeleton dominating the human output and thus resulting in passive walking Hornby et al. (2020).

Despite the fact that the stability analysis for the kinematic and stiffness controllers yields an exponential tracking result, there are inherent factors in the dynamics that influence the walking performance. Hence, the implementation of the treadmill-based walking experiments experience several challenges. The active torque generated by the muscle contractions is influenced by the electromechanical delay (EMD) inherent in the muscle activation dynamics, which degrades joint tracking performance. As depicted in Figure 6 and Figure 7, there exists a muscle contraction delay (i.e., a time difference between the onset of the stimulation and the point when the participant’s muscle force is effectively evoking active force) that affects the response of the muscle during tracking. In practice, input delay influences not only the muscle generated torque but also the response of the electric motors and cable-driven mechanisms. The muscle stimulation response time is within approximately 100–300 ms Downey et al. (2017), which influences the walking tracking performance especially for faster treadmill speeds. Further in Downey et al. (2017), it was concluded for the quadriceps that the EMD increases as the number of muscle contraction increases under isometric conditions. A systematic way to compensate for muscle input delay is to design an input delay compensator to inject a delay-free input in the closed-loop controller, as in previous results Alibeji et al. (2018b). However, a control design to compensate for input delay raises technical challenges to analyze the stability of switched delayed systems, which is a control problem beyond the scope of this paper. Moreover, an estimate of the input delay is likely needed for the effective implementation of the delay-free controller. Muscles experience fatigue that can lead to loss of performance. Similarly to compensating for input delay, the control design can be enhanced to cope with fatigue Alibeji et al. (2018b). Muscle fatigue did not play a major role during the obtained 3-min walking experiments in able-bodied individuals. However, muscle fatigue compensation is needed for individuals with movement disorders who need a high dosage of locomotion training. Asynchronous stimulation patterns such as the ones developed in Downey et al. (2015) can be implemented for assisted walking to lessen the effects of fatigue. Hence, muscle fatigue and delay are important factors to consider for the development of rehabilitative strategies using FES. Moreover, the measurement of the torque about the knee joint using load cells can be prone to noise, which directly affects the quality of the torque tracking objective. Future efforts are to improve the signal quality of the designed torque-based controllers.

From a control perspective, technical improvements in the control design for the muscles and electric motors in the hybrid exoskeleton will be explored. The motivation behind the sliding-mode control terms in Eqs 11, 18 is to compensate for the upper bounds on disturbances and uncertain nonlinearities in the dynamic model and analytically guarantee exponential tracking using a switched system analysis. However, robust control methods exploiting high frequency and high gain can accelerate the onset of muscle fatigue and potentially induce chattering effects. Alternatives to sliding-mode control include using higher-order sliding mode or a continuous version of the sliding-mode controller (e.g., high-slope saturation function using a boundary layer as in Khalil (2002). Further, the Lyapunov-based stability analysis provides conservative, sufficient control gain conditions. Hence, the sufficient gain conditions in Eqs 23, 31 are not necessary. The main challenge to verify the sufficient gain conditions is the lack of exact model knowledge of the muscle dynamics to compute the control effectiveness value for each muscle. Nevertheless, a conservative numerical estimation of the gain conditions can be developed based on a 70-kg participant walking at 0.8 mph. The estimation of human’s segments weight, inertia, and center of gravity, and joint elastic and viscous effects leverages the results in Krishnan et al. (2016); Ferrarin and Pedotti (2000). The muscle effectiveness is estimated under isometric conditions similar to Downey et al. (2015). The conservative, sufficient gain conditions can be numerically estimated to be *k*_2_ ≥ 195.4, *k*_3_ ≥ 28.3, *k*_4_ ≥ 3.9, *k*_5_ ≥ 4.1, *k*_7_ ≥ 2.6, *k*_8_ ≥ 1.6. Due to the conservative bounds, the controllers leverage high gain feedback to cope with the model’s uncertainty. However, implementing large gain conditions as the ones numerically estimated above in real-time experiments is challenging due to the accelerated rate of muscle fatigue *via* FES Downey et al. (2017), hardware performance limits, and control input saturation, which influence the human-machine interaction. Thus, the gain conditions provide guidance to initially select the control gains in Eqs 11, 18 that are subsequently adjusted during experiments to achieve satisfactory tracking performance using an empirical-based approach as described in the Appendix. Therefore, adaptive control methods are desirable to cope with uncertainty through estimation of parametric and non-parametric uncertainty and improve tracking performance, while reducing the need for high-frequency content feedback. Future efforts will examine the control design and stability analysis associated with those novel control alternatives. Motivation also exists to improve the design of torque tracking controllers. The stiffness tracking controller in Eq. 18 uses an auxiliary integral signal of the torque feedback in Eq. 16. Thus, the knee stiffness controller acts as an integral controller, which does not respond instantaneously yet remains bounded as depicted in Figure 10. Despite the slower response of the stiffness integral controller, higher order derivatives of the torque feedback signal are not required for the control design and stability analysis. In fact, the derivative of the torque signal is usually not available for feedback due to noise. Another important control challenge when developing kinematic and stiffness controllers for muscles and motors is their ongoing dynamic interaction during experiments, which raises the need to guarantee stability of both closed-loop error systems. The approach in this paper is to compensate for the interaction terms, but exploring passivity methods Khalil (2002) or energy shaping Lv et al. (2018) can lead to novel human-machine interactions during assisted walking using hybrid exoskeletons. Finally, the desired joint kinematics in this study were generated by exploiting preliminary data collected for each participant. The study of how to optimize the kinematic gait pattern using trajectory optimization methods as in Hereid et al. (2018); Gurriet et al. (2018) are to be explored to customize the trajectories for each individual. Moreover, the developed control methods need to be expanded to account for tracking objectives that do not depend on time but rather on gait phase or a phase-dependent variable (Lv et al., 2018). Time dependent trajectories might not be suitable for walking training of individuals who can apply volition (e.g., stroke survivors) or for locomotion in unstructured environments outside of the laboratory.

The walking performance obtained for the three able-bodied individuals motivates the evaluation of the developed control approach in individuals with different levels of mobility (i.e., participants who require different assistance levels). The integration of lighter devices that minimize resistance with control technology that promotes user’s volition is desired to maximize human effort and intent in individuals with incomplete SCI and strove survivors. It is expected that individuals with SCI could benefit from continuous stepping training at high intensities for a long duration across multiple gait sessions (Hornby et al., 2020). Future work includes the implementation of an active ankle joint orthosis to improve the response and energy efficiency of existing hybrid walking systems.

## 7 Conclusion

Hybrid exoskeletons combine motorized assistance and FES to exploit the benefits of activating paralyzed muscles and the torque reliability of the machine. Kinematic and stiffness tracking controllers were designed and implemented to actuate electric motors and activate lower-limb muscles to achieve treadmill walking at a constant speed. Two walking trials at different speeds were conducted for each of the three participants. A bipedal walking model for the exoskeleton and human is developed using a switched systems approach that captures the transitions for stance to swing phase, and vice versa. For the knee joint, the muscles achieved kinematic tracking and the electric motor achieved the stiffness control objective by adjusting the cable tensions. For the hip joint, the electric motors achieve the kinematic tracking objective. A Lyapunov-based stability analysis is developed to yield exponential tracking for both the kinematic and stiffness closed-loop systems. Control design innovations are required to compensate for muscles input delay in the context of switched systems. Input delay is an important factor that negatively influences walking performance in the hybrid exoskeleton. Validation of the developed methods in individuals with movement deficits will be conducted as part of the future work. Moreover, the development of novel control methods that comply and promote human voluntary effort are desired during gait rehabilitation to achieve a more natural gait pattern in individuals with neurological conditions. Advances in control methods and wearable devices are needed to increase the participant’s gait speed and endurance toward achieving community ambulation after injury.

## Data Availability

The data presented in this study are available on request from the corresponding author.

## Footnotes

1 Surface electrodes for the study were provided compliments of Axelgaard Manufacturing Co., Ltd.

2 The servo drive was provided in part by the sponsorship of Advanced Motion Controls.

## Appendix

### Guidelines for Tuning the Control Gains

Hybrid exoskeletons integrate powered mechanisms and FES to provide assistance and activate muscles during rehabilitative walking. Hybrid exoskeletons aim to improve walking ability and build muscle capacity in individuals with movement deficits. However, the human-exoskeleton dynamics are nonlinear, uncertain and time-varying, which pose technical and practical challenges. Closed-loop controllers are designed in Eqs 11, 18 to overcome these challenges and achieve treadmill walking using the hybrid exoskeleton. To implement the developed controllers in real-time experiments, the practitioner selects control gains that influence the inputs applied to the electric motors and muscles *via* FES. The goal is to adjust the control gains to achieve satisfactory muscle response despite the nonlinear activation rate and time-varying dynamics. The control gains are adjusted for the electric motors to achieve a fast electro-mechanical response without inducing high transients, which can negatively affect the human-robot interaction in particular for individuals with neurological conditions. The control gains introduced in Eqs 11, 18 were tuned during a preliminary trial prior to the actual treadmill walking experiment. During the pretrial for gain tuning, the kinematic controller in Eq. 11 was turned on first, which activates the electric motors that actuate the hip joints and the quadriceps and hamstrings muscle groups. Once satisfactory performance was obtained for the hip and knee kinematic controllers, then the knee stiffness controller was turned on to adjust the response of the electric motors that actuate the knee joint. Additional tuning steps were conducted when both kinematic and stiffness controllers were activated. The selection of the control gains in Eqs 7, 11, 18, 20, 21 is described below.

• *α*: This gain in Eq. 7 adjusts the kinematic controller proportionally to the hip and knee joint angle error. The gain *α* influences the response of the electric motors that actuate the hip joint and the muscles that generate torque about the knee joint. The gain *α* was selected largest among all the control gains to bias the tuning of the control gains towards improving the joint angle kinematic tracking.
• *k*_1_: This gain in Eq. 11 adjusts the kinematic controller by weighting the joint angular position error *e*_*κ*_ and angular velocity error ${\dot{e}}_{\kappa }$ . This gain influences the hip joint electric motors and muscles. This gain was tuned to achieve satisfactory response of the derivative term (i.e., angular velocity) to reach the desired kinematic range of motion.
• *k*_2_–*k*_5_: These gains in Eq. 11 adjust the kinematic controller by weighing the signum function sgn (*r*_*κ*_). The gain *k*_2_ compensates for the constant upper bound in Eq. 10, thus acts as an offset that changes sign. The gain *k*_3_ weighs the norm of the composite error vector *z*_*κ*_, thus acts as a linear term. The gain *k*_4_ weighs the norm squared of *z*_*κ*_ *.* The gain *k*_5_ weighs the norm of the stiffness input, which acts as a cross-term. These control gains are tuned lower compared to the gains *α*, *k*_1_ since their values can amplify nonlinearities and yield chattering effects. As a rule of thumb, *k*_2_ ≥ *k*_3_ ≥ *k*_4_ since they act as constant, linear, and quadratic terms.
• *k*_6_: This gain in Eq. 18 adjusts proportionally the knee stiffness controller by weighing the integral torque error *e*_*s*_ *.* This gain influences the knee joint electric motors. The gain is tuned to balance the stiffness response to prevent a bias to overshoot or undershoot the desired stiffness trajectory.
• *k*_7_–*k*_8_: These control gains in Eq. 18 adjust the knee stiffness controller by weighing the signum function sgn (*e*_*s*_). The gain *k*_7_ weighs the knee kinematic tracking error, thus acts as a proportional term. The gain *k*_8_ weighs the norm of the knee joint kinematic input, which acts as a cross term. These control gains are tuned to reduce the potential chattering effects using force feedback.
• *k*_*e*_: This scaling gain in Eq. 20 adjusts the control command for each electric motor. The gain weighs both the hip kinematic controller and knee stiffness controller.
• *k*_*m*_: This scaling gain in Eq. 21 adjusts the control commands for each muscle (i.e., right and left quadriceps and hamstrings). The gain was increased or decreased to compensate for weaker or stronger muscle responses across the three participants to achieve joint kinematic tracking. In addition, stimulation sensitivity or discomfort was a factor in tuning the muscle gains $k_{m},\forall M$ .
